# Addressing Contemporary Challenges in Pediatric Critical Care Medicine

**DOI:** 10.3390/children13060827

**Published:** 2026-06-18

**Authors:** Balagangadhar R. Totapally

**Affiliations:** 1 Division of Critical Care Medicine, Nicklaus Children’s Hospital, 3100 SW 62nd Avenue, Miami, FL 33155, USA; balagangadhar.totapally@nicklaushealth.org; 2 Herbert Wertheim College of Medicine, Florida International University, Miami, FL 33199, USA

Pediatric critical care medicine has evolved into a highly specialized discipline that continues to adapt to the changing realities of clinical practice [1]. Advances in technology, evolving organ support strategies, progress in oncologic therapies, and increasingly sophisticated regional systems of care have significantly improved outcomes for critically ill children, while also creating new and complex challenges. Pediatric intensivists now care for children with greater medical complexity, navigate difficult decisions around resource utilization and goals of care, and confront persistent disparities in access to specialized services across different healthcare settings [2].

This Special Issue, “Addressing Challenges in Pediatric Critical Care Medicine”, brings together ten articles from countries around the world, reflecting both the global perspective and the broad, evolving scope of contemporary pediatric critical care practice. Collectively, these contributions underscore both persisting challenges—such as sepsis, airway emergencies, trauma, and acute neurologic crises—and emerging priorities, including pediatric transport systems, advance care planning, and the management of increasingly complex chronic conditions.

## 1. Early Recognition and Stabilization

Timely recognition and intervention remain fundamental to modern pediatric critical care. The retrospective study from Türkiye included in this Special Issue highlights pediatric anaphylaxis as a condition in which prompt diagnosis and adherence to evidence-based management protocols are critical for preventing deterioration. Although anaphylaxis is often encountered in emergency settings, variation in recognition and treatment persists, emphasizing the importance of ongoing education and systems-based quality improvement.

Similarly, pediatric acute drug intoxications remain an important and evolving public health concern, and childhood poisoning constitutes a significant global public health challenge [3]. The retrospective analysis from Romania included in this collection reminds us that toxicologic emergencies span both accidental and intentional exposures, requiring age-sensitive prevention strategies, rapid risk stratification, and multidisciplinary management.

Foreign body aspiration is another preventable but potentially catastrophic emergency in children [4]. The review and clinical experience in Bulgaria presented here reinforce the importance of clinical vigilance, caregiver awareness, and timely procedural intervention.

Collectively, these studies from multiple countries highlight a central theme in pediatric critical care: patient outcomes frequently depend on timely recognition, coordinated protocol-driven responses, and robust system preparedness.

## 2. Managing Complex Critical Illness

Several contributions in this Special Issue address conditions characterized by significant physiologic complexity and a high mortality risk. Sepsis remains one of the most challenging pediatric critical illnesses around the world [5]. The narrative review on septic cardiomyopathy in this Special Issue provides an important summary of age-dependent cardiovascular physiology and the distinct hemodynamic responses seen in children. Understanding these developmental differences, as well as the dynamic nature of septic cardiomyopathy, is essential, as pediatric sepsis cannot be approached by simply extrapolating pathophysiology from adult, static models.

Critically ill pediatric oncology patients represent another highly vulnerable and increasing population [6]. Improved cancer survival has increased the number of children with malignancies requiring intensive care support, yet outcomes remain influenced by illness severity, infection risk, treatment-related toxicity, and organ dysfunction [7,8]. The Saudi Arabian single-center experience presented here adds valuable regional data to an area where international experience remains heterogeneous.

Thrombocytopenia in critically ill children, although frequently encountered, often reflects complex and multifactorial pathophysiology. Its presence may serve as both a diagnostic clue and a prognostic marker, reinforcing the importance of integrating laboratory abnormalities into broader clinical assessment rather than treating them in isolation.

These articles collectively illustrate the increasingly nuanced and diverse physiology encountered in modern PICUs.

## 3. Neurocritical Care and Trauma

Neurocritical illness in children continues to challenge clinicians with the need for rapid decision-making in the face of significant diagnostic and therapeutic uncertainty. The narrative review on blood product transfusion and coagulopathy in pediatric traumatic brain injury addresses an area where evidence remains incomplete, and practice varies considerably. Balancing cerebral perfusion, hemostasis, and the risks associated with transfusion remains challenging, particularly in pediatric populations where robust data are limited.

Status epilepticus syndromes similarly demand rapid recognition and escalation of therapy [9]. The review in this collection helps clarify complex, sometimes confusing terminology, especially regarding refractory status epilepticus, while emphasizing practical clinical frameworks relevant to pediatric providers. The authors review the terminology and clinical spectrum of refractory seizure disorders, including refractory status epilepticus, super-refractory status epilepticus, new-onset refractory status epilepticus, febrile infection-related epilepsy syndrome, and refractory status epilepticus associated with various forms of encephalitis, including autoimmune encephalitis.

Together, these articles highlight the ongoing need for pediatric-specific evidence in neurocritical care.

## 4. Pediatric Transport

Pediatric critical care extends far beyond the walls of the intensive care unit. Safe transport of critically ill infants and children remains essential to regionalized pediatric care. The contemporary review of neonatal and pediatric transport included in this collection underscores the importance of coordinated systems, specialized teams, transport physiology expertise, and evolving technology in ensuring continuity of critical care during transfer. This systems perspective is especially important in regions where access to tertiary pediatric intensive care remains uneven. The review emphasizes that transport medicine is not merely a logistical operation but a mobile extension of intensive care.

## 5. Advance Care Planning Communications

Modern pediatric critical care increasingly requires attention not only to physiology but also to communication, family-centered care, and ethical decision-making. Advance care planning in children with refractory oncologic disease represents one of the most emotionally and clinically complex areas of pediatric medicine. The review in this Special Issue emphasizes that meaningful advance care planning is not a single conversation but an evolving process that requires sensitivity, prognostic honesty, and alignment with family values. As survival improves for many serious pediatric conditions, the field must continue to expand its focus from life preservation alone toward quality, dignity, and goal-concordant care.

## 6. Looking Forward

The articles in this Special Issue reflect the remarkable breadth of pediatric critical care medicine from various countries around the world. They span emergency stabilization, organ dysfunction, neurocritical care, toxicology, transport systems, hematologic complications, and communication at the end of life.

A recurring message across these contributions is that pediatric critical care is increasingly multidisciplinary, systems-oriented, and globally relevant. While technology and therapeutics continue to advance, persistent challenges remain: generating pediatric-specific evidence, reducing practice variability, improving equitable access to specialized care, and supporting children with increasing medical complexity.

The future of pediatric critical care will depend not only on scientific innovation but also on thoughtful integration of evidence, systems design, communication, and compassionate family-centered care.

## Data Availability

Not applicable.
